# A Systematic Review of Thirty-One Assessment Tests to Evaluate Mobility in Older Adults

**DOI:** 10.1155/2019/1354362

**Published:** 2019-06-20

**Authors:** Racha Soubra, Aly Chkeir, Jean-Luc Novella

**Affiliations:** ^1^Laboratoire de Modélisation et Sûreté des Systèmes (M2S), Université de Technologie de Troyes, 12 rue Marie Curie, 10004 Troyes, France; ^2^Service de Gériatrie - CHU Reims, Hôpital Maison Blanche, 45 rue Cognacq Jay, 51100 Reims, France

## Abstract

Assessments of gait, balance, and transfer in elderly people play a valuable role in maintaining healthy aging and preventing a decline in mobility. Several evaluation tools have been proposed; however, clinicians should select the most accurate ones wisely, based on numerous criteria. This systematic review aims to identify all applicable elderly mobility assessment tests and show their measurement properties with as much detail as possible. Initially, a broad search was performed. Articles were screened based on their titles and abstracts, and only studies published in English were considered. Based on our inclusion and exclusion criteria, 31 assessment tests evaluating the mobility of healthy elderly people were found. Then, further searches were completed to identify the measurement properties of each test. These characteristics include the origin and year of establishment, several practicality factors, and validity. The analysis of our outcomes illustrates the similarities and differences between the identified tests.

## 1. Background and Purpose

By definition [1], the term “Mobility” has different meanings depending on the context it is used for. In this review, we refer to Mobility as the person's ability to change his position or location or move from one place to another by walking and basic ambulation. Therefore, Mobility is considered as a crucial aspect in order to maintain healthy aging with a good quality of life [2, 3]. In a cohort study of 1128 people aged between 60 and 96 years, an association between elderlies' mobility and the Health-Related Quality of Life (HRQoL) has been demonstrated. Results showed that the ability to walk can lead changes in both physical and mental HRQoL [4]. On the other hand, several physiological and psychological factors can have negative effects on the mobility of older people. For instance, factors like changes in bones, joint problems, muscle weakness, and neurological diseases can lead to mobility impairments [5].

According to World Health Organization (WHO), while the prevalence of musculoskeletal disorders increases with age, some related diseases are the second largest contributor to disability worldwide, such as osteoarthritis, low back problems, hip fracture, sarcopenia, and osteoporosis [3].

Generally, troubles with walking and mobility impairments produce undesirable physical, cognitive, and social consequences for older adults. They often cause a decline in independence, physical disability and injuries, institutionalization, and an increase in hospital admissions [3, 6, 7]. Hence, the activities of daily living, which include a mobility item, start to diminish with age leading to depression, isolation, and death [3]. For this reason, healthcare professionals are keen to recognize subjects who have problems, as well as to determine the type of necessary interventions and their timing in order to plan a better health and healthcare for the aging population [8]. Accordingly, evaluations of mobility are fundamental in gerontology as they identify potential impairments and reduce morbidity. Researchers and specialists refer to mobility measures to (i) identify changes in an individual's mobility, (ii) detect early sign of decline, and (iii) assist in guiding therapeutic interventions [6]. As shown by Van Kan, G.A et al., gait speed could be used as a predictor of adverse outcomes, since it reflects the health and functional status in elderlies [9]. As well, a pooled analysis of individual data collected from 9 cohort studies affirmed the association between gait speed and survival in older adults [10].

Nowadays, many assessment instruments are used to evaluate elderly people's mobility and balance, such as the Timed Up and Go (TUG) test, Short Physical Performance Battery (SPPB), Dynamic Gait Index (DGI), and Berg Balance Scale (BBS). In fact, these tools differ from each other with regard to their functional level, content, and characteristics. Additionally, the interpretation of results could vary from test to another, depending on the methodology of recording outcomes. For instance, some tests analyze quantitative measurements, while others focus on qualitative aspects.

Although there is a lack of consensus on which assessment test to use [11], it is very important to select an accurate one to improve the thoroughness of evaluations, determine precise plans of care, and monitor progress better [8, 12].

The choice will depend on the user's objectives as well as the properties of the tool. In order to choose the appropriate assessment test in the research field and in practice, several factors have to be taken into consideration. Principally, applicable tests must be valid, suitable for the target population, and the practical aspects of their application are well known.

Therefore, the aims of this systematic review were to (i) identify all available and commonly used elderly mobility assessment tests, (ii) point out their content and characteristics, and (iii) summarize their validity when tested on community-dwelling elderly. Our main goal is to provide clinicians and researchers a valuable reference regarding the evaluation of gait, balance, and transfer in elderly people.

## 2. Methodology

Broad research was performed to identify all available and applicable mobility assessment tests for healthy elderly people. Then, further research was performed to summarize the practical content, characteristics, and validity of all available tests as summarized by their founders. In our systematic review, we only looked for articles published in English. However, in order to attain our purposes, the year of publication and the number of citations were not taken into consideration.

### 2.1. Search Strategy

The first objective of our research was to point out all the useful measurement instruments in clinical and research fields which are utilized to analyze the mobility of healthy elderly people. Thus, we aimed to identify the maximum number of available tests and their characteristics. A broad search was conducted using the following databases: Science Direct, Scopus, SAGE, Springer, Wiley, Taylor & Francis Online, and Google Scholar search. An open access to these databases was provided by the University of Technology of Troyes. A manual search was also performed on Google Scholar to identify more relevant references. Our searching methodology included any term or synonym that is related to healthy older adults, geriatric, mobility, and gait evaluation. The list of terms consists of “mobility assessment/test/instrument/evaluation”, “elderly/older/aged people”, and “clinical/geriatric test”. Articles were screened based on their title and abstract. The list of references of the included articles was also scanned in order to identify further information. Once a test was found, we searched for the original article and its developers to assess its validity and main characteristics. All included papers were collected by one author (RS) and examined by two authors (AC and JN).

As explained by VanSwearingen and Branch [5], three major issues should be considered while selecting a measurement tool: (i) appropriateness to the target population, (ii) practical aspects of test administration, and (iii) psychometric properties.

Accordingly, we looked at the preeminent factors for each identified test with the utmost possible detail. For each test, multiple combinations of its name and acronym were used, while using the Google Scholar search. For instance, the conceptual coverage and characteristics of the Six-Minute(s) Walk Test were collected using the following search terms: Six-Minute(s) Walk Test, 6-Minute(s) Walk Test, Six-Minute(s) Walking Test, 6-Minute Walking Test, and 6MWT.

### 2.2. Selection Criteria

The targeted population of this research covers healthy elderly people. Accordingly, an elderly mobility assessment test was excluded if it is solely administered or used to evaluate the mobility of subjects with a specific disease or illness. Furthermore, an article was considered relevant when it tackles general descriptions of an identified measurement test, its main practicality characteristics, and/or its validity.

#### 2.2.1. General Description

In order to clearly present an assessment tool, it is highly significant to gather its general information. Therefore, we aimed to collect data about the origin of a test: its founder(s) and the year of establishment, its main purpose(s): evaluation of gait, measurement of balance, examination of strength and endurance, and so forth and the population for which the test was initially devised (e.g., healthy or frail elderly people).

#### 2.2.2. Practicality Characteristics

It is important to notice that ease of administration is fundamental when choosing a test [8]. For this reason, we looked for articles that pointed out the practicality characteristics of our identified tests. For each assessment test, we searched for (1) the equipment needed for administration, (2) the performance steps (i.e., detailed instructions for the person administering the test and the person being evaluated), (3) whether training or trial tests are required, (4) the format of assessment (i.e., performance-based, judgement-based, or self-report), (5) methods of scoring (e.g., dichotomous scale, 3- or 4-level scale, time records, etc.), (6) whether results are interpreted qualitatively and/or quantitatively, (7) whether it is allowed to use assistive devices or not, and (8) the different versions developed over the years.

#### 2.2.3. Validity

By definition, the concept of validity represents the extent to which a test is measuring what it is supposed to measure. Its evaluation is known to be one of the most important criteria for the quality of a test [13]. Most researchers and clinicians interpret the validity of a measurement to evaluate its accurateness and trustworthiness. However, the determination of validity can be made through four types of measurement depending on the purpose of validation: face validity, content validity, criterion-related validity, and construct validity. Therefore, in this review, we looked for the validity of a test as reported by its founder(s) only. We aimed to summarize the type of study validity, the gold-standard test to which the outcomes of a test were compared, and the correlation coefficients (e.g., Pearson's rho and Spearman's rho) if available.

## 3. Results

### 3.1. Data Collection

A total of 36 elderly mobility assessment tests were found in 2173 articles. However, based on our inclusion and exclusion criteria, only the characteristics of 31 tests were interpreted in this review.

The 5 excluded evaluation tests were used to examine the mobility of elderly patients with traumatic brain injury (High Level Mobility Assessment Tool, HiMAT [14], and Community Balance & Mobility Scale, CB&M [15]), neuromuscular and musculoskeletal conditions (Barthel Index, BI [16]), or neurological disorders such as stroke and multiple sclerosis (Rivermead Mobility Index, RMI [17], and the Modified Emory Functional Ambulation Profile, mEFAP [18]).

**Figure 1:**
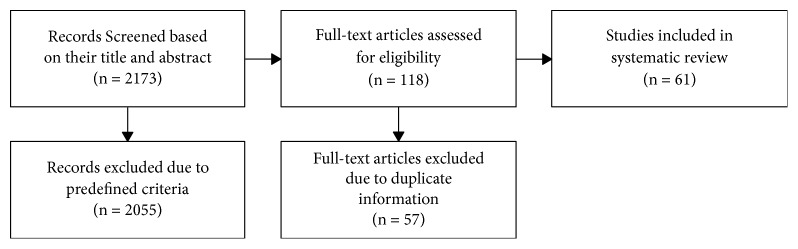
Flow diagram of our review.

The flow diagram, shown in Figure 1, documents our complete literature search.

The 31 selected elderly mobility tests are obviously discussed below and summarized in Tables 1–4. In order to facilitate the selection of a measurement tool, a general description about the acronym, origin, year of establishment, the aim of each test, validity, and the time needed to complete the test have been presented in tables, if available.

Based on our analysis of all measurement scales and their data records, tests were classified in tables depending on their format of assessment. Moreover, they were sorted in descending order according to the number of citations achieved by the founder's article till November 2018.

## 4. Measurement Properties of 31 Mobility Assessment Tests

### 4.1. Performance-Based Measures

#### 4.1.1. Timed Up and Go (TUG)

The Timed Up and Go (TUG) test was firstly described by Podsiadlo and Richardson in 1991 as a modified version of Get Up and Go test [19, 20]. It is a clinical assessment test widely used to assess balance and walking ability in elderly populations [19–21]. To perform this test, participants are observed and timed in seconds, while they rise from an armed chair of approximately 46 cm seat height and 65 cm arm height, walk at their usual pace a distance of 3 meters towards a line marked on the floor, turn 180 degrees, walk back to the chair, and sit down. They are also asked to wear their regular footwear and use their customary walking aid if necessary. The time taken to complete the test is measured by a stopwatch: it commences on the command “go” and ends once the subject's back is positioned against the back of the chair after sitting down. A faster time indicates a better performance. Although it is very simple, the TUG test is highly recommended, since it includes the basic everyday movements and daily life tasks (standing, walking, and turning) and contains valuable components [22, 23]. Moreover, it correlates well with the BBS (r = -0.81), gait speed (r = -0.61), and BI (r = -0.78) [19].

Several modifications have been proposed for this test. For instance, [22] stated a modified TUG version in which participants are asked to walk as fast as they can while ensuring safety. On the other hand, Pernille et al. [24] introduced the Expanded TUG by timing each task separately and using a longer walkway of 10 meters.

#### 4.1.2. Short Physical Performance Battery (SPPB)

Short Physical Performance Battery (SPPB) is an assessment test used to examine gait, balance, strength, and endurance in elderly epidemiological studies and outpatient clinics. Its performance is divided into three subtests: a hierarchical balance assessment, a short usual gait speed test, and 5 Time Sit to Stand (5TSTS) test [25]. First, for balance examination, participants are asked to stand with their feet in a side-by-side, semitandem (the heel of one foot placed to the side of the first toe of the other foot), and tandem (the heel of one foot is directly placed in front of the toes of the other foot) position, consecutively, for 10 seconds, if they are capable. Interviewers stop timing once participants move their feet, grasp for a support, or surpass the 10 seconds. Second, they are timed over a two 8-foot walking course with the use of assistive devices if needed. Analysis is based on the results of the fastest test. Third, participants are asked to perform the 5TSTS test after showing their ability to rise from a straight-backed chair placed next to a wall without using their arms. 5TSTS is another mobility assessment test and is separately discussed below. Scores of each task range from 0 to 4 and are based on the time performance: a task completed in a short time indicates better performance and gives a higher score. As affirmed by Fernando et al. [26], SPPB scores predict a wide range of health consequences such as the ability or disability in Activities of Daily Living (ADLs), loss of mobility, and hospitalization.

#### 4.1.3. Six-Minute Walk Test (6MWT)

Six-Minute Walk Test (6MWT) is a modified version assessment that represents a sensible compromise between 12-minute and 2-minute walking tests [27]. It was initially introduced as an endurance measurement test and has more recently been considered as a general indicator of overall physical performance and mobility in older adults [28, 29]. As declared by Harada et al. [30], although the 6MWT is used to summarize the effect of strength and endurance impairments on walking, it also provides as well information about the functional ability to walk. The test is conducted under a standardized protocol that is used to measure the maximum distance walked on a hard, flat, and hard surface in a period of 6 minutes [27]. Participants are instructed to walk as far as they can in a 100 ft. hallway without running or jogging, and they are allowed to stop and rest during the test. To resume walking, examiners might encourage them by using the two standardized statements only: “You are doing well” and “Keep up the good work”. Additionally, 6MWT assessment includes the global and integrated responses evaluations of several systems involved during exercise such as the pulmonary and cardiovascular systems, blood and peripheral circulations, and muscle metabolism [31]. It has been shown that this test has a high correlation with the 12-minute walk test (r=0.955), 2-minute walk test (r=0.892), and gait speed (r=-0.73) [27, 32].

#### 4.1.4. 8-Foot Up-and-Go (UG)

The 8-Foot Up-and-Go (UG) is a modified version of the TUG test introduced by Rikli and Jones in 1999 as a composite measurement of power, speed, ability, and dynamic balance [33]. It involves the same procedure as the TUG test with slight alterations: the walking distance changes from 9.84 ft. (3 m) to 8 ft. (2.44 m) and the turning phase must be done around a cone instead of a marked line on the floor. The main reasons for these changes were, firstly, to increase the test feasibility by administering it in areas with limited space and particularly in domestic settings and, secondly, to reduce confusion regarding the turning area. After a test demonstration for participants, the UG test must be performed 3 times (1 practice and 2 trials). The instructor will record the time taken to complete each of the 2 trials and select the lowest time as a final score for interpretations.

#### 4.1.5. Usual or Habitual Gait Speed (UGS/HGS)

Walking speed is widely used in research fields as well as in clinical settings as a measurement of gait aspects. It is recognized as an indicator of rehabilitation needs, future functional decline, and fall risk [34]. Thus, the Usual Gait Speed (UGS), also known as Habitual Gait Speed (HGS) or the measurement of a straight path walking velocity, is considered as a useful assessment test that provides significant information about an individual's overall functional capacity. To perform this test, clinicians can refer to various versions of UGS depending on the availability of walking distance (3-, 4-, 6-, and 10 meters walk test, with an additional distance of approximately 5 m for acceleration and deceleration). Participants walk the selected straight-path distance at their comfortable speed without verbal encouragement. According to [35, 36], the 6-meter walk is the most commonly used versions for elderly people studies.

#### 4.1.6. Physical Performance Test (PPT)

Originally described in 1990, the Physical Performance Test (PPT) was developed by Reuben et al. as an assessment tool to monitor and describe through several performance tasks the multiple domains of physical function in frail and well community-dwelling elderly people [37]. These tasks simulate activities of daily living using various degrees of difficulty. Two versions were presented by the developers: a 7-item scale and a 9-item scale, and both demonstrated concurrent validity where high correlation is shown in comparison with basic daily activities and Tinetti-POMA test. The 7-item PPT consists of writing a sentence, simulated eating, turning 360 degrees, putting on and removing a jacket, lifting a book and putting it on a shelf, picking up a pencil from the floor, and a 50 ft. (15.2 m) walk test. The 9-item PPT includes the same items with 2 stair-climbing tasks: time to climb one flight of stairs and number of flights climbed with a maximum up to 4. The majority of PPT items are scored based on the time taken to finish the task. Scores vary from 0 to 28 and from 0 to 36 for the 7-item and 9-item PPTs, respectively, with higher scores showing better performance. PPT involves few instruments and minimal instructions and takes about 10 minutes to complete.

#### 4.1.7. 5-Time Sit-to-Stand (TSTS) Test

The time sit-to-stand (TSTS) is a clinical technique developed by Csuka and McCarty and used to quantify the lower limb strength and muscle force, examine the functional status, and evaluate balance in older adults [38]. This test was originally performed by measuring the time needed to stand up and down 10 times from an unarmed chair while keeping one's arms folded across the chest. Subsequently, the timed chair rise was reduced to five and the test has been known as 5-TSTS test. Despite its apparent simplicity, STS is considered as a sequence of multiple tasks. The ability to go from sitting to standing position reflects an important skill in elderly people. As well, the inability to do the test may lead to institutionalization and impaired function and mobility in activities of daily living [39]. Moreover, it is significant to highlight the importance of STS determinants in evaluating the ability in performing the test. As summarized by Janssen et al. [2] in their review, several STS determinants such as the type of chair, chair seat height, positioning of feet, and the use of armrests may influence the ability of an elder person to do the STS. Thus, neglecting these factors may produce a misleading analysis of the outcomes.

#### 4.1.8. L-Test of Functional Mobility (L-Test)

Deathe et al. conceived a modified version of the TUG test for subjects with lower limb amputation [40]. They proposed a longer walking path, representing an “L” configuration, with a total-covered distance of 20 meters instead of 6-m. This version, entitled L-Test of Functional Mobility (L-Test) incorporates 2 transfers of 3 and 7 meters and 4 turns in both right and left directions. With a similar transfer skill set to the TUG, participants are required to stand up from an armless chair, traverse the L-shape distance of 10 m at their usual selected speed, turn 180 degrees, return the walked distance of 10-m, and sit back down. The time taken to perform this test is recorded using a stopwatch for mobility evaluation. Moreover, developers of this test demonstrated a high correlation between this modified version and TUG (r=0.93), 10-meter walk test (r=0.97), and other measures [40].

#### 4.1.9. Backward Walking (BW)

As declared by Fritz et al. [41] and cited by Middleton et al. [12], the assessment of Backward Walking (BW) may provide clinicians and healthcare professionals additional information about subjects' mobility. Being more sensitive than forward walking, BW helps to predetermine the necessity of fall prevention interventions, the need of assistive devices, and the necessity of assessment intervention efficacy in the elderly [42].

#### 4.1.10. De Morton Mobility Index (DEMMI)

The De Morton Mobility Index (DEMMI), recently developed in 2008, is a validated assessment instrument used to measure the mobility of older adults through clinical settings [43]. It consists of 15 hierarchical items categorized as bed mobility, chair tasks, static balance, gait, and dynamic balance. Eleven items follow a dichotomous scale (0 or 1) and four items are scored from 0 to 2. To calculate the DEMMI score, the total raw score is converted to an interval score out of 100 through Rasch Analysis with higher scores representing better mobility. As explained by Natalie de Morton et al. [1], DEMMI is a safe, quick, and easy to administer unidimensional instrument. The test is conducted in an average of 8 minutes and it only requires a bed or plinth, an arm chair of 45 cm seat height, a pen, and a stopwatch.

#### 4.1.11. Figure of 8 Walk Test (F8W)

The Figure of 8 Walk Test (F8W) was modified in 2010 by Hess et al. in order to characterize the complex walking abilities and skills in everyday life through a combined straight-curved paths [44]. This new test was the first assessment tool to provide curved-path walking consisting of both clockwise and counter clockwise directions, with a straight-path walking between them. Moreover, it has been proven to be a valid measure when compared with gait speed (r=-0.57), GES (r=-0.468), and other balance measurements [44]. To perform this test, the participants are requested to walk a figure-of-8 around 2 cones placed 5 ft. (1.524 m) apart. They have to stand midway between the cones facing outward from the plane of the cones, select the direction of the F8W path, begin walking at their usual selected speed, and stop once they return to the starting position. As outcomes of this test, three skilled movement components are investigated: speed (time to complete the test), amplitude (number of steps taken), and accuracy (F8W completed within 2 ft. of the cones or not). Accordingly, low walking speed, high number of steps, and more than 2 ft. away from the cones show poor performance.

#### 4.1.12. Instrumented Stand and Walk (ISAW) Test

The Instrumented Stand and Walk test (ISAW) is a clinical test used to assess balance and gait in elderly people through synchronized body-worn sensors [45]. These latter consist of wireless Opal™ movement monitors with a 3D angular rate sensor, a 3D accelerometer, and a gyroscope. After putting on the body sensor, participants are asked to stand quietly for 30 seconds and then to walk a distance of 7 meters at their usual pace, turn 180 degrees, and walk back to the initial point. The outcomes of this test combine the measurement of postural sway and anticipatory postural adjustment during step initiation, gait, and turning.

#### 4.1.13. Hierarchical Assessment of Balance and Mobility (HABAM)

In 1995, Macknight and Rockwood have proposed the Hierarchical Assessment of Balance and Mobility (HABAM) instrument [46]. The test aims to display graphically the changes in balance and mobility of hospitalized older adults through mobility, transfers, and balance sections. For each section, a hierarchical range of abilities is constructed. The patient is required to get up from the bed and walk to the best of his ability using his usual walking aid, so he is scored based on his highest score attained. Its construct validity showed a correlation coefficient of 0.76 with BI and 0.74 with BI mobility subscales. In 2000, developers suggested transforming the HABAM instrument from a graphic indicator into a measurement index using Rasch Analysis in order to estimate dimensional intervals [47].

#### 4.1.14. Trail Walking Test (TWT)

Motor cognition, visual performance, and hearing functions are considered as beneficial indices to predict falls. Consequently, Yamada and Ichihashi [48] have introduced the Trail Walking Test (TWT), a test necessitating both cognitive and motor functions in order to be performed successfully. In this test, 15 flags are installed randomly in a 25 m^2^ area at 15 different positions marked with a 30 cm diameter circle, and participants are asked to sequentially move between the numbered flags in an ascending or descending order. The test should be performed only once and timed using a stopwatch.

#### 4.1.15. Parallel Walk Test (PWT)

The Parallel Walk Test (PWT) is an evaluation test devised to assess the dynamic balance and lateral movement for stability of elderly people during ambulation [49, 50]. To perform this test, participants walk a distance of 6 m between 2 parallel lines at their usual normal speed. They are requested to look ahead while walking instead of looking at their foot placement and they are allowed to use their walking stick if needed. The test was initially performed and validated with 3 different randomized widths between the parallel lines (20 cm, 30.5 cm, and 38 cm) and reported an optimal distance of 20 to 30.5 cm to discriminate correctly people with balance problems such as fallers and nonfallers. For scoring, a 1 point will be assigned if the participant places his foot on the line and 2 points if foot placement is outside the line and/or the participant grasps something to maintain balance. Accordingly, a lower score shows better performance.

#### 4.1.16. Charité Mobility Index (CHARMI)

Charité Mobility Index (CHARMI) is a new promising and easy to use test initiated by Liebll et al. at the Charité University Hospital in Berlin, Germany [51, 52]. The test allows the monitoring of mobilization through a set of hierarchical scored items involving positioning, transfers, and locomotion. The items consist of transfers in bed, sitting on edge of bed, transfer from bed to chair, wheelchair mobility, standing, walking, and climbing stairs. During test performance, participants are allowed to use their assisting devices if needed; however, any help or assistance from another person is considered as test failure. Each completed task is pointed by 1, and high continuously achieved score refers to better performance. Although its correlation with the whole BI is low (r=0.63), CHARMI confirmed a concurrent validity with respect to 3 BI items (transfers bed to chair and back, mobility on level surfaces, and stairs) with r=0.93 [51].

#### 4.1.17. Standardized Walking Obstacle Course (SWOC)

The Standardized Walking Obstacle Course (SWOC) is an evaluation test used to assess obstacle negotiation and determine ambulation capacity under three circumstances [53, 54]. One practice and 2 trials are performed for each condition. First, participants are asked to walk along a standardized pathway at their usual speed. Second, they are asked to walk the pathway while holding a tray. Third, they have to perform the test while wearing dark glasses in order to simulate dim-light conditions, such as walking at night. The standardized pathway consists of 12.2 m long (39.5 feet), 0.92 m (36 inches) wide with turns of 30° right, and 70° left and obstacles commonly faced in daily life. SWOC tasks involve standing from a chair A, walking at normal speed towards a chair B, and sitting down and returning to chair A, while avoiding stumbling or stepping off the track. For test evaluation, the examiner measures the time taken to complete the test and counts the number of steps, stumbles, and steps off.

#### 4.1.18. Pick-Up Weight Test

As shown in PPT and BBS, the ability to reach down and pick up an object from the floor is an important task for mobility assessment. In Tiedemann et al.'s study [55], this task is considered as a single mobility evaluation test for community-dwelling elderly.

### 4.2. Performance- and Judgement-Based Measures

#### 4.2.1. Tinetti Performance-Oriented Mobility Assessment (POMA)

The Tinetti Performance-Oriented Mobility Assessment (Tinetti-POMA), also known as Tinetti Mobility Test (TMT), is a clinical test used to measure balance and gait in elderly people. It was originally devised in 1986 by Tinetti and consisted of 13 balance tasks and 9 items for gait assessments in order to predict falls in an institutionalized population [56]. Later on, a modified and commonly used version has been introduced. It reduced the examination into 9 balance tasks (POMA-B) including sitting, rising from a chair, attempting to rise, immediate standing, standing with eyes opens and standing with eyes closed, sternal nudge, turning 360°, and sitting down, plus 7 items to assess gait characteristics (POMA–G) consisting of initiation of gait, step length and height, step symmetry, step continuity, path, trunk stability, and walking stance. Each task is scored on a 2-point or 3-point scale. Scores are combined providing a maximum total score (POMA-T) of 28 points with subtotal score of 16 and 12 points for POMA-B and POMA-G, respectively. A total score less than 19, varying between 19 and 24 and varying between 25 and 28, represents, respectively, high (abnormal), medium (normal), and low (adaptive) risk of fall. Over time, new Tinetti-POMA versions have been used with some modifications in the items performance and scoring procedures [8]. They are widely used in various clinical contexts as a measurement of mobility impairment and studies of the effects of interventions [57].

#### 4.2.2. Berg Balance Scale (BBS)

Developed in 1989, the Berg Balance Scale (BBS) is a measurement tool used to assess balance in elderly people [58]. At first, the test involved 38 balance tasks. Later, it has been refined to combine 14 items that are executed in clinical settings. These items consist of a variety of functional positions such as transfers, sitting unsupported, standing with eyes closed and feet together, picking up objects, and placing alternate foot on a stool amongst others. The test evaluation is based on the ability of a participant to perform the tasks independently in a minimal time and/or to reach a specific distance without external support or assistance. Each item scores from 0 to 4, giving a maximum total score of 56 with higher scores indicating better performance.

#### 4.2.3. Dynamic Gait Index (DGI)

The Dynamic Gait Index (DGI) was developed in 1997 by Shumway-Cook et al. in order to examine the functional stability of elderly people during gait activities and evaluate their risk of falling [59]. The test consists of 8 items that are used to evaluate a person's response to change when following the clinician's demand while ambulating. The functional tasks include walking a distance of 50 ft. (15.2 m), walking while changing gait speed, walking with head turned in the vertical and horizontal directions and walking with pivot turn when announced, stepping over and around obstacles, and ascending/descending stairs. Each item is scored from 0 to 3 points, giving a maximum total score of 24 points. Higher score shows better functional mobility and balance stability. Later on, a faster version consisting of 4 items was introduced to give similar information as the 8-item DGI [60].

#### 4.2.4. Balance Evaluation Systems Test (BESTest)

The balance Evaluation Systems Test (BESTest) is performed as a clinical balance assessment tool [61]. The test consists of 36 items performed under 27 tasks and aims to evaluate 6 different balance control systems: biomechanical constraints, stability limits/verticality, transitions/anticipatory postural adjustments, postural responses, sensory orientation, and stability in gait. Each item is rated on a 4-level scale where 0 points and 3 points refer to the worst and best performances, respectively. Consequently, a percentage of the total point is obtainable for the total score and for each section as well. The evaluation of this test takes approximately 30-45 minutes. Therefore, two shortened versions have been introduced in order to improve the BESTest clinical utility and feasibility: Mini- and Brief-BESTest. With variations in the literature, the Mini-BESTest involves 14 versus 16 items rated on 3-point scale [62], and the Brief-BESTest involves 6 versus 8 items rated on 4-point scale [63, 64]. Each of these shortened versions takes approximately 10 to 15 minutes for performance.

#### 4.2.5. Functional Gait Assessment (FGA)

The Functional Gait Assessment is an ambulation-based balance test based on DGI test and initially proposed to assess the functional stability in individuals with vestibular disorders [65]. It shows an acceptable concurrent validity in comparison with other gait and balance measures [65]. The test includes 7 of the 8 items presented in DGI with 3 additional tasks: walking a distance of 20 ft. (6 m) with narrow base of support (tandem stance), walking backward and walking with eyes closed, and the 7th DGI item (walking around obstacles) is not included.

#### 4.2.6. Alternate Step Test (AST)

The Alternate Step Test (AST) is an adjusted version of the stool-stepping task available in the BBS [55, 58]. The test aims to measure lateral stability, assess clinical balance, and predict fall risk. It involves participants alternatively placing their entire right and left foot 8 times as quickly as possible on a step/stool of approximately 18 cm, rather than just touching the stool as in BBS stool-stepping task.

#### 4.2.7. Elderly Mobility Scale (EMS)

Elderly Mobility Scale (EMS) test was developed by Rachael Smith in 1994 to assess mobility in the frail elderly [66]. The test examines transfer, gait, and balance through the evaluation of seven functional activities of daily living: lying to sitting, sitting to lying, sitting to standing, standing, gait, 6 meters-time walked, and functional reach. Each item is scored on a 2-point, 3-point, or 4-point scale and scores are summed to provide a final total score that varies between 0 (totally dependent mobility) and 20 (independent mobility). A total score under 10, between 10 and 13, and above 14 represents, respectively, “dependence in mobility manoeuvres”, “borderline in terms of safe mobility”, and “likely to be independent in mobility” [66, 67]. EMS test reports a high concurrent validity with BI (r=0.962) and the functional independence measure (r=0.948) [66].

#### 4.2.8. Physical Performance and Mobility Examination (PPME)

The Physical Performance and Mobility Examination (PPME) is an observed-administered test developed in 1990 as a measurement instrument of the physical functioning and mobility of hospitalized older people [67, 68]. It involves the evaluation of 6 items: bed mobility, transfer skills, multiple stands from a chair, standing balance, step-up ability, and ambulation with walking aids if needed. These tasks take approximately 10 minutes to be performed and necessitate the availability of a bed, chair, stopwatch, and standardized step. The outcomes of each item are scored on either a pass-fail scale (0 or 1 point) or a three-level scale (high pass/2 points, low pass/1 point, or fail/0 points) giving a maximum total score of 6 or 12, respectively. Its construct validity suggests that PPME can add a unique dimension of mobility [68].

#### 4.2.9. Functional Obstacle Course (FOC)

In 1996, Kevin Means developed the Functional Obstacle Course (FOC) as a rehabilitation setting tool [69]. The test aims to evaluate elderly subjects with balance and mobility dysfunction while performing 12 simulated functional tasks usually faced in and over the home environment. The FOC stations challenge the physiologic strategies in balance and ambulation by means of four stations with different types of floor surfaces; two ramps; two sets of stairs; and four discrete functional tasks (opening and closing a door, rising from a chair, walking a linear distance of approximately 106 m, and stepping over foam cylinders). Qualitative (the quality of performance, need of assistance, and apparent difficulties) and quantitative (the time taken to perform the course) outcomes are analyzed, giving a maximum total score of 36. In order to eliminate the need for parallel bars and/or prevent obstacles interchange, a modified and valid FOC version was created by placing some obstacles next to walls [70]. This version correlates significantly with gait velocity, 6MWT, and Tinetti-POMA.

#### 4.2.10. TURN180 or TURN360

As previously shown, turning tasks appear in several mobility assessment tests such as TUG, BBS, and Tinetti-POMA. However, as explained by Simpson et al., this task is a measure in its own right [71]. It is known as an evaluation technique of dynamic postural stability in elderly frail people particularly for those with complex problems [71]. Some examiners use the 180° turn and others the 360° turn test. For instance, in TURN180 test, participants are required to step around 180 degrees without grasping for assistance or using walking aids. A detailed protocol for this test is available in [71]. Quantitative and/or qualitative outcomes are evaluated depending on the selected version. Examiners mainly report the time taken to complete the test and count the number of steps while turning. As shown in Wang et al.'s study [72], detailed and more accurate analysis is accessible due to recent technologies such as video sensors and cameras. Recently, Kobayashi et al. devised a new assessment test of 180 degrees standing turn strategy (CAT-STS) in order to evaluate turning while standing in various elderly populations [73].

#### 4.2.11. Duke Progressive Mobility Skills Test

As cited by Duncan et al, Duke Progressive Mobility Skills is a mobility assessment test developed by Hogue et al. in 1990 [74–76]. It consists of 13 items evaluating static and dynamic balance such as sitting, rising, chair transfer, walking at usual and maximal speed, stepping over obstacles, and climbing stairs. Each item is scored on a 3-point scale: 0, unable to complete the task or assistance is required; 1, task performed but abnormally; and 2, task performed normally. Hence, a higher total score indicates better performance.

### 4.3. Self-Report Measures

#### 4.3.1. Life Space Mobility Assessment (LSMA)

The life space measurement was initially introduced by May et al. in 1985 [77]. They initiated the Life Space Diary (LSD) in which participants will record the zones they moved to during each day over a period of 1 month. In order to document their mobility within their home and community, the traveled zones are divided into 5 areas: the bedroom, the rest of the dwelling, the yard or grounds surrounding the dwelling, the neighborhood, and the area across a traffic-bearing street. Accordingly, the life space mobility assessment (LSMA) evaluates mobility based on how far and how often a person transfers to the defined zones with or without assistance. It shows what participants actually did rather than what they were capable of doing [78] (i.e., it reflects the actual performance of mobility activities in daily life and tracks if changes occurred). LSMA studies are based at the University of Alabama in Birmingham (UAB), Study of Aging Life-Space Assessment [79]. Scores vary between 0 (totally bed-bound) and 120 (traveled out of town every day without assistance).

#### 4.3.2. Modified Gait Efficacy Scale (mGES)

Gait Efficacy Scale (GES) is a mobility evaluation test based on the principle of self-sufficiency in walking. As conceived, an individual's perception of his or her walking ability plays an important role in mobility evaluation. Accordingly, GES aims to recognize the confidence of an elderly person in performing challenging gait tasks. A modified GES (mGES) version has lately been introduced in order to add items more often encountered in everyday walking [32]. This version showed an association with several mobility performance tests such as TUG, 6MWT, F9W, gait speed, and obstacle course tests. To perform the mGES, participants are asked to rate their confidence about performing each of the 10 walking tasks individually. Tasks include walking on a level surface, walking on grass, walking safely over obstacles, stepping up and down from a curb, ascending and descending stairs safely (with and without a handrail), and walking over a long distance. Each item is scored on a 10-point Likert scale, with 1 representing no confidence and 10 representing complete confidence, thus giving a total score ranging between 10 and 100.

## 5. Discussion

To the best of our knowledge, this is the first systematic review that points out a plethora of mobility assessment tests. We identified 31 tests that are used to evaluate gait, transfer, and balance of healthy elderly people.

The main objectives of our review were to summarize all available mobility assessment tests and show their characteristics with the utmost precision. We aim to provide clinicians and researchers with valuable knowledge about the mobility measurement tools to enable them to select the correct one wisely.

As revealed before, mobility is crucial for getting through the day and enjoying healthy aging. Accordingly, several evaluation tools have been devised in order to prevent and/or treat the loss of mobility in the community-dwelling elderly. However, the challenge remains in determining the appropriate measurement test based on numerous criteria. Several considerations in clinical sensibility must be studied, such as purpose and target, content validity, ease of usage, suitability of scale, and overt format.

The first step involves setting the main purpose(s) behind the mobility assessment of an elderly subject. In our view, we believe that a mobility assessment test should be chosen based on whether it was initiated to satisfy a targeted purpose or not. A test that is developed for a certain aim could not be applied to accomplish another task. Clinicians and researchers may seek to attain different purposes behind their examination. For instance, in 1986, Tinetti suggested that mobility evaluations aim at identifying components of mobility difficulty related to performing daily activities, knowing the reasons for difficulty throughout specific tests, and determining possible health risks caused by immobility. On the other hand, Kishner and Guyatt explained that assessment tests, clinical measurements, and social science can be used for three purposes: to discriminate between subjects, to predict results (prognosis), and to evaluate changes over time. It was also remarkable that mobility could be analyzed through three major fields: evaluation of gait, balance, and transfer. Accordingly, it is highly important to outline the domains required to be analyzed. In this review, a general description regarding the purpose(s) of each identified test has been afforded. Particularly, we can notice that 8 tests were developed to evaluate gait only, 12 tests were initiated to evaluate gait and balance, and 7 tests aimed to assess gait, balance, and transfer in the elderly population. However, 5TSTS, FR, pick up weight tests, and AST were the only measurements used to assess endurance, balance, and transfer.

Although it is crucial to choose a measurement based on the initial purpose(s) of evaluation, several factors should be wisely examined and investigated as well.

Under Feinstein's proposition [80], the qualitative characteristics of a test represent a significant property to determine how acceptable a test is. For instance, a magnificently valid test with a difficult index of use (e.g., huge number of items, expensive equipment, necessity of space, etc.) will not be selected by a clinician. Numerous aspects can facilitate the decision-making and help in selecting the appropriate one(s).

VanSwearingen and Brach [8] explained three major issues that should be appraised: (1) appropriateness to the target population, (2) practicality, and (3) psychometric properties. Therefore, we can deduce that, at first, a selected measurement tool must have been previously tested on a group of people similar to the people to be evaluated. Then, the choice of a tool needs to reflect a reasonable practicality. This latter refers to a set of factors that may intervene in the test selection; it includes the time needed to administer and perform a test, the necessary equipment, the method of scoring, the format of assessment, and the format of interpretation of results. For instance, an objective measurement that necessitates a long time of performance and a lengthy subjective measurement may lead to the fatigue of an older adult. On the other hand, a costly space required and/or inaccessible equipment may be difficult to achieve.

Moreover, three major clinimetric properties are defined to be the key indicators of the quality of any measurement instruments or tests [13]: validity, reliability, and responsiveness. In principal, a valid and reliable test refers to the extent to which this test is measuring what it purports to measure and is free from measurement error, respectively. However, the responsiveness of a test refers to its ability in detecting a change over time in the construct of interest.

Furthermore, it is worth noting that measurement techniques are generally classified into two methods: subjective and objective measurements. Subjective measures also known by proxy's methods are based on a person's perception. The results of these methods are often obtained through questionnaire, surveys, or interviews. On the other hand, objective measures are observer-rated instruments. The outcomes rely on a participant's performance of a test and an observer's evaluation. In order to facilitate the selection procedure, we have categorized the identified mobility assessment tests into three formats: (i) performance-based measurement: referring to a test in which participants accomplish it and generate a ratio score, (ii) judgment-based measurement: referring to a test in which observers/raters score the test based on their examination, and (iii) self-report measurement: referring to a questionnaire answered by the participants [8]. As investigated in Guralnik et al.'s study [81], each of these formats has its advantages and disadvantages. Accordingly, researchers and clinicians have to make a compromise between several aspects according to their required purposes. For instance, they can refer to subjective measures in order to cover a variety of topics in a brief amount of time and with a reduced administrative cost. However, it should be known that, in this case, outcomes may be inaccurate as participants could overestimate or underestimate their mobility performance and capabilities. However, this does not confirm that objective measures are superior or interchangeable with subjective measures. Both types of measures have their own impact in the evaluation of a functional status. A self-report measurement could provide information about the functional status of a person that cannot be obtained by an objective measure and vice versa. Accordingly, many researches support the associations between both formats as both have strengths and limitations [82, 83]. Nevertheless, the choice of a measurement type relies on the objective of the evaluation. Decisions differ from a clinician or researcher to another with reference to their study and purposes.

In order to facilitate the selection process, this systematic review gathers the mobility assessment tests and categorizes them according to their type of measures. It was remarkable that the majority of elderly mobility assessment tests belong to objective measures (performance-based and/or judgement-based measurements). From the 31 identified tests, the LSMA and mGES were the only self-report measures.

Moreover, several additional pragmatic criteria need to be addressed. However, we notice that numerous terms were used to define an instrument as ideal, such as “applicability, acceptability, feasibility, practicality, usefulness/utility, availability, and so forth”. Auger et al. suggested classifying pragmatic criteria under the umbrella of applicability and grouped them into four categories (respondent burden, examiner burden, score distribution, and format compatibility) [84]. This variety in terms and definitions created confusion in defining the valuable aspects that could be used for selecting the most appropriate mobility assessment test. Accordingly, we looked for the maximum available information and characteristics for each test. We gathered information about the administration time and equipment, the complexity and simplicity of a test, the detailed instructions for participants and observers, the results scoring, and methods of interpretation if available. Our findings show that the majority of tests allow the use of assistive devices and do not require much equipment for administration. However, the major differences that appear between tests depend on the main purpose of evaluation, results interpretation, and the time and space needed for performance.

It was also remarkable that mobility outcomes could be interpreted differently. Most of the tests seek to interpret quantitative outcomes; however, few tests seek to interpret both quantitative and qualitative outcomes. Quantitative outcomes are mostly based on the time taken to complete a test and the maximum walked distance. On the other hand, qualitative outcomes are based on the observer's evaluation and the test performance. In our opinion, as both interpretations enclose strengths and weaknesses, none of quantitative and qualitative approaches could be considered as superior or inferior to the other.

An additional issue could intervene in the selection of the appropriate test and should be taken into consideration: “the care setting”. This latter affects the time, space, and equipment needed for a test performance. For example, we can notice that 9 tests (6MWT, HABAM, DGI, FGA, PPT, DEMMI, CHARMI, SWOC, and FOC) require more space than the other tests and could not be supervised in a small clinic or at home.

Regarding the psychometric properties, we have simply reported the validity of tests as first declared by their founders. Although the gold-standard test is not yet acknowledged, the founders of 14 tests reported high correlation coefficients outcomes and proved the validity of their devised measurement tests. It is our intention to review the reliability and responsiveness later.

Last but not least, we believe that any selected measurement should be appropriate to the target population. When evaluating the mobility of an elderly subject, the test must be chosen if it is initially developed or previously used with people similar to the target subject. In this review, our targeted population was the healthy elderly people. We identified mobility assessment tests that are not used for condition specific elderly (e.g., stroke patient, Alzheimer's subject). Nevertheless, we believe that several factors can alter the way a person walks. Although Balzac's Theory of Walking was written in a sarcastic style with a hint of irony, a scientific and erudite way was offered to describe the human gait and to discuss factors influencing gait [85]. Balzac admitted that weight, height, personality, occupation, social standing, either race or weather, and other psychological factors can influence gait. Additionally, as declared in the study of Holmes and Holmes [86], the world is made up of different cultures; subsequently aging experiences appear at different scales. Thus, we can admit that seniors growing in some country have a walking pattern they go through which may not be identical or similar to those of other elderlies ageing in other societies or countries. For instance, WHO launched a longitudinal study to examine the gait speed at different phases of age in six different countries (China, Ghana, India, Mexica, Russian Federation, and South Africa). Although the time needed to walk 4 meters increases with age, it is worth remarking that the values of this increment differ between the countries [87, 88].

Nevertheless, in most of mobility assessment tests, the interpretation of results is built on the concept of comparing outputs with a certain reference scale. For example, an elderly subject who accomplishes the Tinetti-POMA test with a total score less than 18 points is considered to have a high risk of falls. As well, these reference scales could be based on the “vital signs of walking” of a group of elderly people who performed the test under specific conditions. Accordingly, it seems valuable to deliberate the factors influencing gait into the reference scale. Involving such references could facilitate the selection procedure and help attain accurate results.

To conclude, since a wide list of mobility assessment tests exist, a summary table could be helpful to serve as a consumer's guide. As declared by McDowell in 1987 [89], “A universal perfect index can never exist”. It is impossible to imagine a single measurement tool to be suitable for all diseases, all individuals, and all applications. Thus, providing adequate information for clinicians and researchers is crucial to achieve standardization and sensibility. As shown previously, several methodological classifications between the measurement tests exist. Accordingly, a reference guide, provided in Table 5, has been proposed to show the distinguishable information about the purpose, targeted population, and settings of each test. All mentioned tests (31 mobility measurements) are applicable for healthy condition of older adults and geriatric care. However, some of the tests could also serve in evaluating the mobility of older adults with stroke, spinal injuries, Parkinson's Disease, back pain, and other diseases.  Table 6 summarizes the applicable mobility test for the major diseases.

## 6. Conclusion

This review summarizes existing measurements that are used to evaluate the mobility of healthy elderly people. A clear description of every tool was provided. It affords a general information set about each measurement test, followed by their important practicality characteristics and validity outcomes if available. Accordingly, clinicians and researchers can more easily find the information necessary to select a form of assessment based on their needs and the purpose of their study.

## Figures and Tables

**Table 1:** Performance-based measures for elderly mobility assessment.

N	Test	Origin	Number of Citations	Aim of Evaluation	Time of administration
4.1.1	Timed Up and Go (TUG)	Podsiadlo & Richardson (1991)	9583	Assessment of balance & walking ability	< 15 minutes

4.1.2	Short Physical Performance Battery (SPPB)	Guralnik et al. (1994)	5224	Examination of gait, balance, strength & endurance	10 to 15 minutes

4.1.3	Six-Minute Walk Test (6MWT)	Butland et al. (1982)	1604	Evaluation of physical performance, endurance & mobility	Up to 6 minutes

4.1.4	8 Foot-Up-and-Go (UG)	Rikli & Jones (1999)	1556	Measurement of power, speed, ability & dynamic balance	Easily & safely

4.1.5	Usual/Habitual Gait Speed (UGS or HGS)	Van Kan et al. (2009)	1068	Evaluation of gait speed	Dependent on the specified walking distance

4.1.6	Physical Performance Test (PPT)	Reuben et al. (1990)	831	Monitoring multiple domains of physical function in frail & community-dwelling elderlies	< 10 minutes

4.1.7	5-Time Sit-to-Stand (5TSTS)	Csuka & McCarty (1985)	668	Evaluation of lower limb strengths, muscle forces, balance & functional status	In a short time

4.1.8	L-Test of Functional Mobility (L-Test)	Deathe et al. (2005)	137	Initially initiated to evaluate gait of subjects with lower limb amputation	10th of a second

4.1.9	Backward Walking (BW)	Laufer, Y (2005)	123	Evaluation of mobility sensitively	Dependent on the specified walking distance

4.1.10	De Morton Mobility Index (DEMMI)	De Morton et al. (2008)	115	Measurement of elderly mobility through clinical settings	Average of 8.8 minutes (SD 3.9)

4.1.11	Figure of 8 Walk Test (F8W)	Hess et al. (2010)	103	Characterize the complex walking abilities & skills	Minimal time

4.1.12	Instrumented Stand & Walk Test (ISAW)	Mancini et al. (2014)	84	Assessment of balance & gait through synchronized body-worn sensors	Quick protocol

4.1.13	Hierarchical Assessment of Balance & Mobility (HABAM)	Macknight & Rockwood (1995)	67	Graphical display of balance & mobility changes over time through mobility, transfer and balance evaluations	Longitudinal study

4.1.14	Trail Walking Test (TWT)	Yamada & Ichihashi (2010)	37	Evaluation of gait through motor cognition & motor function	Easily made

4.1.15	Parallel Walk Test (PWT)	Lark et al. (2009)	27	Assessment of dynamic balance & stability during ambulation	Quick test

4.1.16	Charité Mobility Index (CHARMI)	Liebl et al. (2011)	3	Monitoring of mobilization through positioning, transfers & locomotion	Up to 15 minutes

4.1.17	Standardized walking Obstacle Course (SWOC)	Taylor et al. (1997)	2	Evaluation of mobility capacity under circumstances & ability to negotiate obstacles	Not found

4.1.18	Pick-Up Weight Test	Tidemenann et al. (2008)	353	Assessment of the ability to reach down and pick up an object	Few seconds

**Table 2:** Performance- and judgement-based measures for elderly mobility assessment.

N	Test	Origin	Number of Citations	Aim of Evaluation	Time of administration
4.2.1	Tinetti Performance Oriented Mobility Assessment (Tinetti-POMA or TMT)	Tinetti (1986)	3191	Measurement of balance & gait	10 to 15 minutes

4.2.2	Berg Balance Scale (BBS)	Berg et al. (1989)	2318	Assessment of balance & transfers in elderly people	15 to 20 minutes

4.2.3	Dynamic Gait Index (DGI)	Shumway-Cook et al. (1997)	1080	Evaluation of functional stability & fall risks	15 minutes

4.2.4	Balance Evaluation Systems Test (BesTest)	Horak et al. (2009)	553	Evaluation of 6 different balance control systems	May take up to 40 minutes for diseased people

4.2.5	Functional Gait Assessment (FGA)	Wrisley et al. (2004)	357	Evaluation of functional stability & fall risks	5 to 10 minutes

4.2.6	Alternate Step Test (AST)	Tiedemann et al. (2008)	353	Assessment of balance & lateral stability	Few seconds

4.2.7	Elderly Mobility Scale (EMS)	Smith et al. (1994)	182	Evaluation of gait, transfer & balance through functional activities	15 minutes

4.2.8	Physical Performance & Mobility Examination (PPME)	Winograd et al. (1990)	149	Evaluation of the physical functioning and mobility of hospitalized elderly	Approximately 10 minutes

4.2.9	Functional Obstacle Course (FOC)	Kevin Means (1996)	75	Evaluation of balance & mobility dysfunction	274.6 ± 131.2 seconds for faller & non-faller

4.2.10	TURN180	Simpson et al. (2002)	35	Evaluation of dynamic postural stability	Few seconds

4.2.11	Duke Progressive Mobility Skills Test	Hogue et al. (1990)	34	Assessment of mobility through static & dynamic balance evaluation	Approximately 10 minutes

**Table 3:** Self-report measures for elderly mobility assessment.

N	Test	Origin	Number of Citations	Aim of Evaluation	Time of administration
4.3.1	Life Space Mobility Assessment (LSMA)	May et al. (1985)	122	Evaluation of mobility based on how far and how often a person transfers to 5 selected zones	On a period of 1 month

4.3.2	Modified Gait Efficacy Scale (mGES)	Newell et al. (2012)	30	Evaluation of mobility based on self-efficacy in walking	Less than 5 minutes

**Table 4:** The validity of 14 mobility assessment tests as firstly declared by their founders.

Test	Population	Reference Test (correlation coefficient)
Timed Up and Go (TUG)	60 patients (mean age of 79.5 years)	(i) Berg Balance Test (BBS) (r= -0.81) (ii) Gait Speed (r= -0.61) (iii) Barthel Index (r= -0.78)

6-Minute walk test (6MWT)	30 patients (mean ± SD age of 61±12 years)	(i) 12-minute walk test (r= 0.955) (ii) 2-minute walk test (r= 0.892) (iii) Gait Speed (r= -0.73)

Physical Performance Test (PPT)	183 elderly subjects from 6 patient populations	(i) self-reported measures of physical function (high correlation (0.50 to 0.80)) (ii) self-reported health status, mental health & cognitive status (moderately correlated (0.24 to 0.47))

L-Test	93 people with unilateral amputations (mean of 55.9 years)	(i) walk test (high correlation) (ii) self-report measures (fair to moderate correlation)

De Morton Mobility Index (DEMMI)	older acute medical inpatients	(i) Barthel Index & HABAM Test (Significant & high correlation)

Figure of 8 Walk (F8W)	51 community dwelling elderly with mobility disability	(i) Gait Speed (Pearson rho= -0.57) (ii) Late-Life Function and Disability Instrument (Pearson rho= -0.47) (iii) Gait Efficacy Scale (GES) (Pearson rho= -0.468)

Hierarchical Assessment of Balance & Mobility (HABAM)	28 patients aged 65 years and older	(i) Barthel Index (r=0.76) (ii) BI mobility subscales (r= 0.74)

Parallel Walk Test (PWT)	27 elderly fallers & 34 elderly non-fallers	(i) Tandem stance (significant correlation)

Charité Mobility Index (CHARMI)	87 subjects from acute care rehabilitation	(i) Barthel Index (r= 0.63) (ii) 3 mobility items in Barthel Index (r= 0.93)

BesTest	22 subjects with & without balance disorders (aged between 50 to 88 years)	(i) ABC Scale (r= -0.636)

Functional Gait Assessment (FGA)	6 patients with vestibular disorders (mean ± SD age of 58.7±12.4 years	(i) Timed Up and Go (TUG) (r= -0.50) (ii) Dynamic Gait Index (DGI) (r= 0.80)

Elderly Mobility Scale (EMS)	36 patients aged between 70 & 93 years	(i) Barthel Index (Spearman's rho = 0.962) (ii) Functional Independence Measure (Spearman's rho = 0.948)

Physical Performance & Mobility Examination (PPME)	498 subjects with impaired mobility (>=65 years old)	(i) activity of daily living (ADL) (r=0.70) (ii) instrumental activity of daily living (iADL) (r=0.43) (iii) Mini-Mental State Examination (MMSE) (r= 0.36)

Modified Gait Efficacy Scale (mGES)	102 community-dwelling older adults (mean ± SD age of 78.6±6.1 years)	(i) Performance based mobility (r=_.38 –.64) (ii) Late-Life Function and Disability Instrument (r=_.32–.88) (iii) measures of confidence and fear (r=_.54 –.88)

**Table 5:** Comparison of the 31 mobility assessment tests used for healthy older adults and geriatric care.

N	Test	Purpose	Format of Assessment	Equipment	Time of administration
4.1.1	Timed Up and Go (TUG)	Assessment of balance & walking ability	Performance-Based	Armed chair of approx. 45 cm seat height and 65 cm arm height and Stopwatch	< 15 minutes

4.1.2	Short Physical Performance Battery (SPPB)	Examination of gait, balance, strength & endurance	Performance-Based	8-ft walkway, Unarmed chair, and Stopwatch	10 to 15 minutes

4.1.3	Six-Minute Walk Test (6MWT)	Evaluation of physical performance, endurance & mobility	Performance-Based	100 ft. hallway and Stopwatch	Up to 6 minutes

4.1.4	8 Foot-Up-and-Go (UG)	Measurement of power, speed, ability & dynamic balance	Performance-Based	Armed chair, Stopwatch and Cone	Easily & safely

4.1.5	Usual/Habitual Gait Speed (UGS or HGS)	Evaluation of gait speed	Performance-Based	Hallway and Stopwatch	Dependent on the specified walking distance

4.1.6	Physical Performance Test (PPT)	Monitoring multiple domains of physical function in frail & community-dwelling elderlies	Performance-Based	Book, Pencil, Paper, Shelf, Stairs and Jacket	< 10 minutes

4.1.7	5-Time Sit-to-Stand (5TSTS)	Evaluation of lower limb strengths, muscle forces, balance & functional status	Performance-Based	Unarmed chair and Stopwatch	In a short time

4.1.8	L-Test of Functional Mobility (L-Test)	Initially initiated to evaluate gait of subjects with lower limb amputation	Performance-Based	L-shape walkway, Chair and Stopwatch	10th of a second

4.1.9	Backward Walking (BW)	Evaluation of mobility sensitively	Performance-Based	No need of equipment	Dependent on the specified walking distance

4.1.10	De Morton Mobility Index (DEMMI)	Measurement of elderly mobility through clinical settings	Performance-Based	Bed, Plinth, Armed chair of 45 cm seat height, Pen and Stopwatch	Average of 8.8 minutes (SD 3.9)

4.1.11	Figure of 8 Walk (F8W) Test	Characterize the complex walking abilities & skills	Performance-Based	2 cones and Stopwatch	Minimal time

4.1.12	Instrumented Stand & Walk (ISAW) Test	Assessment of balance & gait through synchronized body-worn sensors	Performance-Based	Opal™ movement with 3D angular rate sensor, 3D accelerometer and Gyroscope	Quick protocol

4.1.13	Hierarchical Assessment of Balance & Mobility (HABAM)	Graphical display of balance & mobility changes over time through mobility, transfer and balance evaluations	Performance-Based	Walkway, Bed and Stopwatch	Longitudinal study

4.1.14	Trail Walking Test (TWT)	Evaluation of gait through motor cognition & motor function	Performance-Based	25 m^2^ area, 15 flags, Marker and Stopwatch	Easily made

4.1.15	Parallel Walk Test (PWT)	Assessment of dynamic balance & stability during ambulation	Performance-Based	Marked line on floor	Quick test

4.1.16	Charité Mobility Index (CHARMI)	Monitoring of mobilization through positioning, transfers & locomotion	Performance-Based	Bed, Chair, Stairs and Wheelchair	Up to 15 minutes

4.1.17	Standardized walking Obstacle Course (SWOC)	Evaluation of mobility capacity under circumstances & ability to negotiate obstacles	Performance-Based	Hallway, Chair, Dark glasses and Tray	Not found

4.1.18	Pick-Up Weight Test	Assessment of the ability to reach down and pick up an object	Performance-Based	Object of approx. 5 Kg	Few seconds

4.2.1	Tinetti Performance Oriented Mobility Assessment	Measurement of balance & gait	Performance & Judgement Based	15 ft. walkway, Armless chair, and Stopwatch	10 to 15 minutes

4.2.2	Berg Balance Scale (BBS)	Assessment of balance & transfers in elderly people	Performance & Judgement Based	15 ft. walkway, 2 Standardized Chair (with and without arm rests), Obstacles, and Step/Stool	15 to 20 minutes

4.2.3	Dynamic Gait Index (DGI)	Evaluation of functional stability & fall risks	Performance & Judgement Based	Walking hallway, Stopwatch, Obstacle and Stairs	15 minutes

4.2.4	Balance Evaluation Systems Test (BesTest)	Evaluation of 6 different balance control systems	Performance & Judgement Based	Stopwatch, 10 degrees incline ramp, Stair step (15 m in height), Obstacles, Chair and Tape	May take up to 40 minutes for diseased people

4.2.5	Functional Gait Assessment (FGA)	Evaluation of functional stability & fall risks	Performance & Judgement Based	Walking hallway, Stopwatch and Stairs	5 to 10 minutes

4.2.6	Alternate Step Test (AST)	Assessment of balance & lateral stability	Performance & Judgement Based	Step/Stool of 18 cm height approximately	Few seconds

4.2.7	Elderly Mobility Scale (EMS)	Evaluation of gait, transfer & balance through functional activities	Performance & Judgement Based	Hallway, Bed, Chair, Yardstick and Stopwatch	15 minutes

4.2.8	Physical Performance & Mobility Examination (PPME)	Evaluation of the physical functioning and mobility of hospitalized elderly	Performance & Judgement Based	Bed, Chair, Stopwatch and Standardized step	Approximately 10 minutes

4.2.9	Functional Obstacle Course (FOC)	Evaluation of balance & mobility dysfunction	Performance & Judgement Based	4 stations with different types of floor, 2 ramps, 2 sets of stairs, Chair, Foam cylinders and Stopwatch	274.6 ± 131.2 seconds for faller & non-faller

4.2.10	TURN180	Evaluation of dynamic postural stability	Performance & Judgement Based	No need of equipment	Few seconds

4.2.11	Duke Progressive Mobility Skills Test	Assessment of mobility through static & dynamic balance evaluation	Performance & Judgement Based	Chair, Walkway, Obstacles, Stairs and Stopwatch	Approximately 10 minutes

4.3.1	Life Space Mobility Assessment (LSMA)	Evaluation of mobility based on how far and how often a person transfers to 5 selected zones	Self-Report	No need of equipment	On a period of 1 month

4.3.2	Modified Gait Efficacy Scale (mGES)	Evaluation of mobility based on self-efficacy in walking	Self-Report	Obstacles, Stairs with handrail and Curb	Less than 5 minutes

**Table 6:** Different populations and their corresponding mobility evaluation tests.

Population	Mobility Assessment Test
Stroke	TUG, 5TSTS, BBS, Tinetti, PPT, BesTest, DGI, FGA, 6MWT, UGS
Spinal Injuries	UGS, LSMA, 6MWT, Tinetti, TUG, BBS
Parkinson's Disease	Turn 180, PPT, BesTest, BBS, BW, DGI, FGA, Tinetti, UGS, TUG, 5TSTS
Osteoarthritis	5TSTS, TUG, BBS
Alzheimer's	TUG
Vestibular Disorder	5TSTS, BesTest, DGI, FGA, TUG, BBS
Multiple Sclerosis	BesTest, 6MWT, DGI, UGS, 5TSTS, Tinetti
Arthritis	5TSTS
Back Pain	5TSTS
Neuromuscular Disease	UGS
